# Microcephaly Prevalence in Infants Born to Zika Virus-Infected Women: A Systematic Review and Meta-Analysis

**DOI:** 10.3390/ijms18081714

**Published:** 2017-08-05

**Authors:** Antonio Victor Campos Coelho, Sergio Crovella

**Affiliations:** 1 Department of Genetics, Federal University of Pernambuco, Avenida da Engenharia, Cidade Universitária, Recife 50740-600, Brazil; avccbio@gmail.com; 2 Institute for Maternal and Child Health, Scientific Institute for Research, Hospitalization and Care (IRCCS) Burlo Garofolo, 34137 Trieste, Italy; 3 Department of Developmental and Reproductive Sciences, Genetic Unit, University of Trieste, 34127 Trieste, Italy

**Keywords:** flavivirus, arbovirus, congenital Zika virus syndrome, microcephaly, emergent diseases

## Abstract

Zika virus is an emergent flavivirus transmitted by *Aedes* genus mosquitoes that recently reached the Americas and was soon implicated in an increase of microcephaly incidence. The objective of the present study is to systematically review the published data and perform a meta-analysis to estimate the prevalence of microcephaly in babies born to Zika virus-infected women during pregnancy. We searched PubMed and Cochrane databases, included cohort studies, and excluded case reports and case series publications. We extracted sample sizes and the number of microcephaly cases from eight studies, which permitted a calculation of prevalence rates that are pooled in a random-effects model meta-analysis. We estimated the prevalence of microcephaly of 2.3% (95% CI = 1.0–5.3%) among all pregnancies. Limitations include mixed samples of women infected at different pregnancy times, since it is known that infection at the first trimester is associated with higher risk to congenital anomalies. The estimates are deceptively low, given the devastating impact the infection causes over children and their families. We hope our study contributes to public health knowledge to fight Zika virus epidemics to protect mothers and their newborns.

## 1. Introduction

The Zika virus infection is an emergent disease that gathered renewed international attention since 2015. This flavivirus was discovered in 1947 in the Zika Forest (present day Uganda) where it was circulating in monkeys and *Aedes* genus mosquitoes. Slowly, the virus spread to Asia and Oceania, causing small outbreaks. However, in 2007 a major outbreak was detected in Yap, a Micronesian island, where over 70% of the population was infected [1].

Later, the virus was introduced in South and Central America through human migration. Evidence points that it was probably introduced between mid-2013 or early-2014 in Rio de Janeiro, Brazil, perhaps favored by international sports events, such as the Football Confederations Cup (June 2013) and Football World Cup (June 2014), which attracted travelers from all around the world [2].

In Brazil, the Zika virus partnered *Aedes aegypti* as its main mosquito vector, and spread through autochthonous transmission to almost all states of the country, until its first detection in the country in May 2015. Estimates suggest that between 500,000 and 1.5 million people have been infected in Brazil [3]. It soon became a concerning national epidemic, following the suspicion that it could cause brain damage and microcephaly in fetuses, because an apparent 20-fold increase in microcephaly incidence was observed amongst an outbreak of an exanthematic disease (later recognized as Zika virus epidemics) in Northeast Brazil [4,5].

The virus was detected in fetal brain tissue and amniotic fluid supporting the virus’ mother-to-child transmission and a causal role on microcephaly occurrence [6]. Brazil is undoubtedly the most affected country by the ongoing Zika virus epidemics. Up until June 2016, 8165 suspected microcephaly cases were registered in Brazil. Among those, 3466 were discarded, 1638 confirmed and 3061 were still in ongoing investigation. Most of the confirmed cases were in the northeast region (1471 of 1638 cases, 89.8%) [7] of Brazil.

The available evidence suggest that infection during the first trimester of pregnancy, in which the fetus’ central nervous system is being formed, is associated with higher risk of microcephaly, miscarriages, and perinatal death occurrence. Additionally, not much else is known regarding the natural history of Zika virus infection [8]. For example, to our knowledge, the prevalence of babies affected by the virus is not well established. Therefore, we aimed to systematically review published data and perform a meta-analysis to estimate the prevalence of microcephaly in babies born to Zika virus-infected women during pregnancy and, therefore, contribute with public health information and decision-making for the management of Zika virus infections and their consequences.

## 2. Results

### 2.1. Selected Studies and Their Characteristics

**Figure 1:**
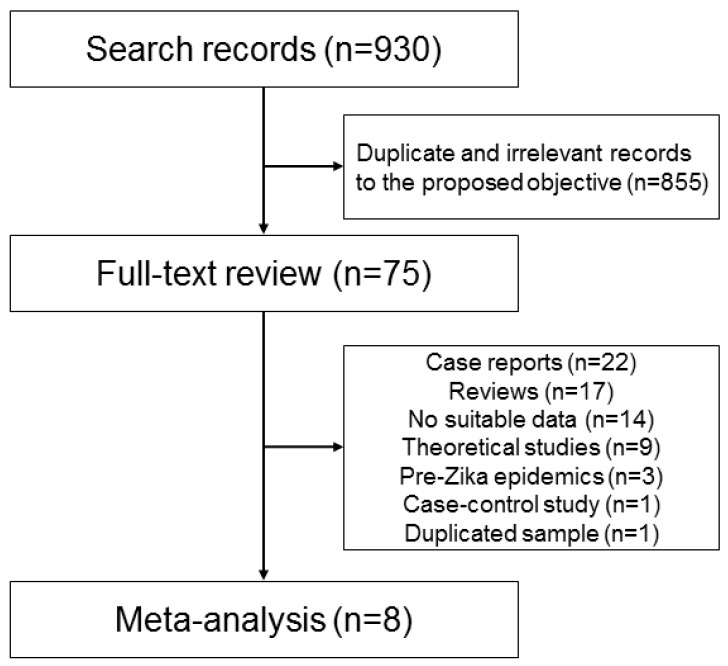
Flowchart of studies selected for review and inclusion on meta-analysis modeling.

Our systematic review search strategy produced 930 abstracts, from which 75 unique abstracts seemed to be relevant for our proposed objective. However, after full-text review, only eight studies [9,10,11,12,13,14,15,16] were included in the meta-analysis, which are summarized in Table 1. The remaining 67 studies were removed because 22 were case reports, case series, or cross-sectional studies, 17 were review or commentary studies, 14 had no suitable data for extraction, nine were theoretical studies regarding Zika virus infection population dynamics, three were investigation of microcephaly prevalence pre-Zika epidemics, one was a case-control study and one study (with different focus) was performed with the same sample of a study included in the meta-analysis [11], so we removed it to avoid data duplicity. Figure 1 is a flowchart of studies selection. Supplementary Materials Table S1 contains the answers for the critical appraisal skills programme (CASP) checklist lists, whereas Supplementary Materials Table S2 lists the removed studies and the motive for their removal.

Overall, the studies detected Zika virus infection through real-time PCR when possible, immunoglobulin M (IgM) testing, and plaque reduction neutralization test (PRNT), following protocols similar to Center for Disease Control’s recommendations [17]. The number of infected women varied between 0.2 [16] and 52.8% [11] of the recruited/assessed women, and one study exclusively recruited Zika virus-infected women [13].

The studies provided slightly different microcephaly definitions. Some studies defined microcephaly as head circumference at least three standard deviations smaller than expected for the infant age and sex [10,15], whereas other studies used two standard deviations as the threshold [11,14] or head circumference lower than the third percentile expected for age and sex [9,13,16]. Microcephaly definition was not identified in a single study [12]. The inclusion criteria for pregnant women also varied slightly among studies. They were in their late twenties [13,14,16] or early thirties [11], on average.

The women recruited by the studies were infected in different trimesters. Some studies recruited mostly women infected during the first trimester [9], whereas others were recruited mostly from the second trimester [11] but, most commonly, there was a complex mixture of probable infection times [13,14,15,16]. It was not possible to assess in which trimester of Zika virus infection possibly occurred in two studies [10,12]. Regarding symptom presentation, the studies reported symptom occurrence frequency as little as 17.2% [16] and as high as 56% [15] or more [9], and the most common symptoms were fever, skin rashes, or joint pain. No significant statistical correlation was observed for proportion of infected women and microcephaly prevalence (data not shown).

Few studies among the eight reported co-infection analyses, with approximately 26.0% (33/127) of the Zika virus-infected women with history of dengue virus infection [11] and a prevalence of 0.3% (1/301) [14] of cytomegalovirus and toxoplasmosis co-infections, or other known teratogenic agents [18].

The authors observed varying degrees of Zika virus-associated microcephaly, with values as low as of 0.3% (four microcephaly cases in 1484 live-birth pregnancies) [10] and as high as 14.3% (one microcephaly case in seven live-birth pregnancies) [9]; and two studies did not detect any microcephaly cases in their samples [12,16]. The frequency of infant deaths (miscarriages and perinatal deaths) varied between 3.0 (two cases in 67 pregnancies) [12] and 22.2% [9]. Most studies did not report secondary outcomes. Two studies reported a prevalence of eye damage around 1.0% [13,14] and cardiovascular damage around 1.0%, as well [14].

A study reported a prevalence of “neural tube defects and early brain malformations eye anomalies, or consequences of CNS dysfunction without brain anomalies or microcephaly” of around 2% [15] and another reported a prevalence of around 7.0% of genitourinary tract, liver, and spleen anomalies detected by an ultrasound exam [14]. The studies’ characteristics are also summarized in Table 1.

### 2.2. Meta-Analyses Results

**Figure 2:**
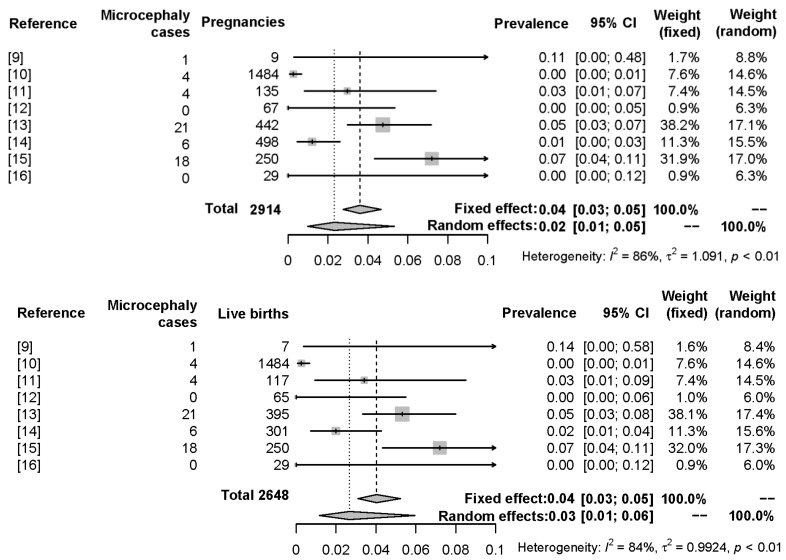
Forest plot of the prevalence of the outcomes in the meta-analysis models 1 (**top**), which deals with the Zika virus-associated microcephaly prevalence among all pregnancies observed by the studies, and 2 (**bottom**), which deals with the Zika virus-associated microcephaly prevalence among live births only for the same studies. The plot displays pooled sample size (2914 pregnancies, model 1, or 2648 live births, model 2), individual prevalence estimates by each study, pooled prevalence estimate (fixed and random effects models), corresponding 95% confidence intervals, study weighting (fixed and random effects models), and heterogeneity measures.

With a total sample size of 2941 mother-infant pairs (pregnancies), 2648 being live births, the random effects model provided a pooled (averaged) prevalence of Zika virus-associated microcephaly of 2.3% (model 1; 95% CI = 1.0–5.3%). When considering the live births, the pooled estimate was 2.7% (model 2; 95% CI = 1.2–6.0%). Due to sample size variability, heterogeneity was high among the studies, always above *I*^2^ = 80% for both models. The meta-analysis results are summarized on Table 2, and Figure 2 displays forest plots for both models.

## 3. Discussion

A sudden and concerning rise in microcephaly cases in Northeast Brazil prompted several investigations that provided geographic, temporal, and molecular evidence that linked the Zika virus with congenital anomalies [19]. The Zika virus epidemics are still unfolding, but key information about the natural history of the infection is still lacking, such as how likely it is that Zika virus infection will affect a pregnancy.

Therefore, we performed a systematic review of studies investigating the consequences of Zika virus infection during pregnancy and conducted meta-analysis modeling to estimate the prevalence of microcephaly.

We estimated the prevalence of microcephaly as being between 2.0 and 3.0%, depending on the stratification used (2.3% considering all completed pregnancies and 2.7% considering only live births).

Our estimates seem to be close to theoretical estimates published previously. A retrospective study on French Polynesia, the setting of one of the first concerning Zika virus outbreaks (occurring between October 2013 and April 2014), estimated a Zika virus-associated microcephaly prevalence of 0.95% [20], slightly below our estimates’ 95% CI lower bounds. Interestingly, a modeling study using reports from Puerto Rico yielded the same estimate: 2.3% of infants born to Zika-infected women could be born with microcephaly [21]. It is important to note that a recent Brazilian study estimated a case fatality rate of 8.3% (171 deaths among 2063 confirmed cases of microcephaly) [22]. The authors also point that if new confirmed cases are included (“cases that would be confirmed if the current 30% confirmation rate of microcephaly cases remained the same when all the cases would have completed the investigation” as defined by the authors) in the analysis, the fatality rate would be 5.7% [22].

It was estimated that, in the French Polynesian outbreak, over 60% of the population was infected [20]. Other studies simulated different seroprevalence rates. A study using data from Bahia, a Northeastern Brazilian state, showed that a rate of 10.0% of Zika virus infection in the general population would be expected to produce a prevalence of 13.2% of microcephaly, slightly below our estimates. On the other hand, if the infection rate was 80.0%, the expected microcephaly prevalence would be 13.2%, above our estimates [23]. Another study using data from Brazil observed that the most affected states, such as Paraíba and Pernambuco, the prevalence was estimated to be 17.1% and 2.5%, considering a Zika virus seroprevalence rate of 50% [24].

The seroprevalence among the recruited women by the reviewed studies seem to be plausible, since rates as high as 73% have been observed [25], but we did not observe a correlation between the proportion of infected women with microcephaly prevalence during our statistical analyses with the aggregated data.

Our estimated prevalence for Zika virus-associated congenital anomalies (microcephaly) seems to be low. However, we must not consider this estimate alone. First, as mentioned above, Zika virus has a very high attack rate; in other words, it is capable of infecting a substantial proportion of the population, which can harbor a number of pregnant women, therefore exposing several children to the risk of life-limiting congenital anomalies or even early death in as much as 8.3% of the affected [22], which bring additional psychological and economic burden to families in areas already struggling with social welfare, such as Northeastern Brazil, the most affected by the unfolding epidemic.

Second, we may compare them to the baseline frequency of microcephaly. In Brazil, for example, a recently-published survey observed a rate of 1.98 microcephaly cases per 10,000 live births per year (0.02%) [24]. In Europe, estimates point to 1.53 per 10,000 live births (0.02%) [26] and in the USA, 8.7 per 10,000 live births (0.09%) [27]. Therefore, Zika virus infection would be, in theory, associated with ten times higher risk (on average) to microcephaly occurrence, with studies reporting figures such as 18–127-fold higher probability of microcephaly with a seroprevalence rate of 50% [24]. A preliminary case-control study observed that the odds of a Zika virus-infected mother having a child with microcephaly was at least 8.6 times when compared with non-infected mothers [28].

Our study has some limitations. First, the reviewed studies recruited a mixture of women infected during different pregnancy trimesters. Since the first trimester is a critical period for the development of the central nervous system [8], our estimates may have suffered some bias, but this is quite difficult to assess. It is important to note that they are close to other published estimations. Second, microcephaly is just one consequence along a spectrum of damages elicited by the virus in the developing fetuses. Third, Zika virus infection is an emerging disease, therefore, not all countries are equally equipped, mostly due to a lack of specific financial support, to fight the disease; this possibly influenced in the rates of conclusive microcephaly diagnostics and hospital/child healthcare infrastructure. These factors, in turn, could be responsible for the observed differences among numbers of symptomatic women (100% in a USA study [9] versus 17% in French Guiana [14]) and rates of fetal survival, which arguably led to wide confidence intervals.

We chose microcephaly as the primary outcome because it drew wide public and research attention, so we assumed that our search strategy would yield a wealth of studies to be appraised. In reality, there is evidence that some infants with apparently normal-sized heads endured severe brain damage, such as the three fetuses with head circumferences in the normal range at birth, but had severe ventriculomegaly in a recent study, and this stresses “how Zika virus infection can be missed if only newborns with microcephaly are assessed”, in the authors words [29]. Therefore, it is possible that some infants “escaped” recruitment and were not included in the studies searched, underestimating the true rate of brain damage caused by the virus. Moreover, microcephaly is just one of several consequences of congenital Zika virus infection. We suggest our readers to consider reading the studies listed in Supplementary Materials Table S2. They were not included in the meta-analysis due to our study design choices, but they report invaluable information regarding Zika virus consequences and organ damage in children.

## 4. Materials and Methods

### 4.1. Systematic Review: Literature Search Strategy and Study Selection Criteria

We searched studies investigating Zika virus infection association with microcephaly through the PubMed database and MeSH (Medical Subject Headings) using the following keywords: (“Zika Virus Infection" [Mesh]) and “Microcephaly/statistics and numerical data” [Mesh]; Zika microcephaly prevalence; congenital Zika syndrome; Zika pregnancy outcome; USZPR microcephaly; ZAPSS microcephaly; Zika microcephaly Brazil; Zika virus microcephaly rate and zika virus microcephaly fatality rate. We also searched Cochrane database of Systematic Reviews with the Zika virus keyword to check if other reviews with similar objectives have been published.

We defined microcephaly prevalence as the ratio between the number of observed cases of microcephaly and the number of recruited pregnant women. Thus, we screened the titles and abstracts of each search result to check for relevance to the proposed objective (to estimate the prevalence of microcephaly, as mentioned earlier).

Following this first assessment, the full-text of the possibly-relevant articles was then further reviewed to check eligibility for the inclusion in the meta-analysis. They were selected if they (1) were cohort studies involving pregnant women and their children and (2) with extractable data since the primary investigated outcome was microcephaly prevalence, the number of recruited women-infant pairs (sample size) and absolute or percentage number of microcephaly cases had to be clearly reported for the inclusion. Thus, case reports or case series studies were not selected for further analysis, because they typically do not provide suitable data for prevalence calculations. We extracted secondary data, such as the number of miscarriages and perinatal deaths (defined as the ratio between the number of deaths and number of all recruited pregnancies) and reports of non-central nervous system organs damage.

All data were independently extracted by each author and registered in standardized electronic spreadsheets. Additionally, each critically reviewed the quality of the studies by answering the CASP cohort study checklist [30]. The authors unified the data, resolving any inconsistencies or omissions before further analysis. To assess risk of bias in outcomes classification, we reviewed the definition of microcephaly cases provided by each report alongside women inclusion criteria, their age and trimester of pregnancy at the time of the study and frequencies of symptoms presentation and other viruses co-infection, such as dengue virus and chikungunya virus.

### 4.2. Meta-Analysis Strategy

We chose a random effects model because it involves an assumption that the effects being estimated in the different studies are not identical [31]. In the random effects model, the variability among the observed prevalence of microcephaly caused by the Zika virus infection is, therefore, attributed to both within-study factors (sampling error for example) and between-study variance (differences in population genetics, Zika virus seroprelavence, and so on). Fixed effect model [32] results are presented as a reference, but all interpretation will be using the random effects model results.

Briefly, microcephaly prevalence estimates (proportions) were log-transformed for the pooled estimation through inverse-variance weighting method [31] and back transformed to simple proportions alongside 95% confidence intervals (95% CI) for the interpretation of results. Heterogeneity between studies’ sample sizes was measured by the τ^2^ statistic and classified by an I^2^ measure (≤25%, between 25 and 50%, between 50 and 75% and between 75 and 100% were considered as low, moderate, high, and very high heterogeneity, respectively) and was evaluated by Cochran’s Q test with *n* − 1 degrees of freedom (in which n is the number of studies included and with significance level α = 0.10 for this test) to check if it was significantly different from zero.

Two meta-analysis models were performed, with the same methodological configurations, to reflect different data stratification patterns: (model 1) microcephaly prevalence among all pregnancies (live births and miscarriages and perinatal deaths), and (model 2) microcephaly prevalence among live births only.

Both meta-analyses were performed through the “meta” package [33] for R software, version 3.4.0 (R Core Team, Vienna, Austria) (April 2017) [34].

## 5. Conclusions

Our meta-analysis following a review of the literature of Zika virus infection of pregnant women yielded estimates for the occurrence of microcephaly in newborn infants that are deceptively low, given the devastating impact that the epidemics had, and is still having, over several families in Brazil and other affected countries in the Americas. It is fundamental to continue research on the Zika virus, so treatments and vaccines are developed, to protect countless numbers of our mothers and children exposed to the unfolding epidemics. Our study confirms previous findings and contributes with public health knowledge and may help the elaboration of public policies regarding Zika epidemics.

## Figures and Tables

**Table 1:** Summary of studies’ characteristics.

Reference	Country	Enrolled Pregnant Women	Zika Virus-Infected Pregnant Women	Proportion of Symptomatic Women ^a^	Live Births	Loss to Follow-up	Microcephaly Cases	Deaths ^b^	Ocular Damage	Cardiovascular Damage	Other Organs Damage
[9]	USA	258	9	100.0%	7	0	1	2	NA	NA	NA
[10]	Colombia	11,984	1484	NR	1484	0	4	0	NA	NA	NA
[11]	Brazil	345	135	27.0%	117	9	4	9	NA	NA	NA
[12]	Puerto Rico	9343	67	100.0%	65	0	0	2	NA	NA	NA
[13]	USA	442	442	38.0%	395	0	21	47	0.9%	NA	NA
[14]	French Guiana	1690	498	17.0%	301	177	6	20	1.0%	1.0%	7.0%
[15]	USA	1297	250	56.0%	250	0	18	0	NA	NA	2.0%
[16]	USA	14,161	29	17.0%	29	0	0	0	NA	NA	NA

^a^, Any of the most common symptoms: fever, skin rash and joint pain; ^b^, miscarriages and perinatal deaths; NR, not reported by the authors; NA, not assessed by the authors.

**Table 2:** Meta-analysis results summary.

Model	Outcome, Stratification	Pooled Estimate (Prevalence)	95% Confidence Interval	Heterogeneity		
				τ^2^	*I*^2^	Q ^a^, *p*-Value
1	Microcephaly, all pregnancies (pooled *n* = 2914)	2.3%	1.0–5.3%	1.09	85.6%	48.6, <0.001
2	Microcephaly, live births (pooled *n* = 2648)	2.7%	1.2–6.0%	0.99	84.3%	44.7, <0.001

^a^, Cochran’s Q test for heterogeneity with seven degrees of freedom.

## Supplementary Materials

Supplementary Table S1 contains the answers for the CASP checklist lists, whereas Supplementary Table S2 lists the removed studies and the motive for their removal. Supplementary materials can be found at www.mdpi.com/1422-0067/18/8/1714/s1.
