# Supplementary material for: Microcephaly Prevalence in Infants Born to Zika Virus-Infected Women: A Systematic Review and Meta-Analysis
Source: Int J Mol Sci. 2017 Aug 5;18(8):1714. doi: 10.3390/ijms18081714 (PMC5578104; doi:10.3390/ijms18081714)
Supplement: Supplementary file 1 [file ijms-18-01714-s001.zip › Supplementary Table 2.docx]

Supplementary Table 2. Studies not included in the meta-analysis and the reasons for their exclusion.

| **Reference** | **Reason for exclusion** |
| --- | --- |
| [1] | review/commentary |
| [2] | case report, case series, or cross-sectional studies |
| [3] | no suitable data for extraction |
| [4] | no suitable data for extraction |
| [5] | review/commentary |
| [6] | no suitable data for extraction |
| [7] | review/commentary |
| [8] | case report, case series, or cross-sectional studies |
| [9] | theoretical study regarding Zika virus infection population dynamics |
| [10] | case report, case series, or cross-sectional studies |
| [11] | review/commentary |
| [12] | microcephaly pre-Zika epidemics |
| [13] | no suitable data for extraction |
| [14] | no suitable data for extraction |
| [15] | no suitable data for extraction |
| [16] | case report, case series, or cross-sectional studies |
| [17] | case-control study |
| [18] | case report, case series, or cross-sectional studies |
| [19] | review/commentary |
| [20] | case report, case series, or cross-sectional studies |
| [21] | theoretical study regarding Zika virus infection population dynamics |
| [22] | case report, case series, or cross-sectional studies |
| [23] | case report, case series, or cross-sectional studies |
| [24] | review/commentary |
| [25] | theoretical study regarding Zika virus infection population dynamics |
| [26] | case report, case series, or cross-sectional studies |
| [27] | review/commentary |
| [28] | review/commentary |
| [29] | different study using same sets of patients of another study |
| [30] | theoretical study regarding Zika virus infection population dynamics |
| [31] | theoretical study regarding Zika virus infection population dynamics |
| [32] | no suitable data for extraction |
| [33] | review/commentary |
| [34] | case report, case series, or cross-sectional studies |
| [35] | review/commentary |
| [36] | case report, case series, or cross-sectional studies |
| [37] | microcephaly pre-Zika epidemics |
| [38] | no suitable data for extraction |
| [39] | case report, case series, or cross-sectional studies |
| [40] | case report, case series, or cross-sectional studies |
| [41] | review/commentary |
| [42] | case report, case series, or cross-sectional studies |
| [43] | review/commentary |
| [44] | microcephaly pre-Zika epidemics |
| [45] | theoretical study regarding Zika virus infection population dynamics |
| [46] | review/commentary |
| [47] | no suitable data for extraction |
| [48] | review/commentary |
| [49] | theoretical study regarding Zika virus infection population dynamics |
| [50] | review/commentary |
| [51] | theoretical study regarding Zika virus infection population dynamics |
| [52] | theoretical study regarding Zika virus infection population dynamics |
| [53] | case report, case series, or cross-sectional studies |
| [54] | no suitable data for extraction |
| [55] | review/commentary |
| [56] | no suitable data for extraction |
| [57] | review/commentary |
| [58] | no suitable data for extraction |
| [59] | case report, case series, or cross-sectional studies |
| [60] | case report, case series, or cross-sectional studies |
| [61] | case report, case series, or cross-sectional studies |
| [62] | case report, case series, or cross-sectional studies |
| [63] | case report, case series, or cross-sectional studies |
| [64] | case report, case series, or cross-sectional studies |
| [65] | case report, case series, or cross-sectional studies |
| [66] | no suitable data for extraction |
| [67] | no suitable data for extraction |

**List of references**

1. Alvarado, M.G.; Schwartz, D.A. Zika Virus Infection in Pregnancy, Microcephaly, and Maternal and Fetal Health: What We Think, What We Know, and What We Think We Know. *Archives of Pathology & Laboratory Medicine* **2016**, *141*, arpa.2016-0382-RA.

2. Aragao, M.F.V.V.; Brainer-Lima, A.M.; Holanda, A.C.; van der Linden, V.; Vasco Aragão, L.; Silva, M.L.M.; Sarteschi, C.; Petribu, N.C.L.; Valença, M.M. Spectrum of Spinal Cord, Spinal Root, and Brain MRI Abnormalities in Congenital Zika Syndrome with and without Arthrogryposis. *AJNR. American journal of neuroradiology* **2017**.

3. Barcellos, C.; Xavier, D.R.; Pavão, A.L.; Boccolini, C.S.; Pina, M.F.; Pedroso, M.; Romero, D.; Romão, A.R. Increased hospitalizations for neuropathies as indicators of Zika virus infection, according to health information system data, Brazil. *Emerging Infectious Diseases* **2016**, *22*, 1894-1899.

4. Besnard, M.; Eyrolle-Guignot, D.; Guillemette-Artur, P.; Last??re, S.; Bost-Bezeaud, F.; Marcelis, L.; Abadie, V.; Garel, C.; Moutard, M.-L.; Jouannic, J.-M.*, et al.* Congenital cerebral malformations and dysfunction in fetuses and newborns following the 2013 to 2014 Zika virus epidemic in French Polynesia. *Eurosurveillance* **2016**, *21*, 30181.

5. Bhadelia, N. Prospective cohort study of pregnant Brazilian women elucidates link between Zika virus infection and fetal abnormalities. *Evid Based Med* **2016**, *21*, 1-1.

6. Buekens, P.; Alger, J.; Althabe, F.; Bergel, E.; Berrueta, A.M.; Bustillo, C.; Cafferata, M.-L.; Harville, E.; Rosales, K.; Wesson, D.M.*, et al.* Zika virus infection in pregnant women in Honduras: study protocol. *Reproductive Health* **2016**, *13*, 82.

7. Byass, P.; Wilder-Smith, A. Utilising additional sources of information on microcephaly. In *The Lancet*, 2016; Vol. 387, pp 940-941.

8. Carvalho, M.D.C.G.; Miranda-Filho, D.d.B.; van der Linden, V.; Sobral, P.F.; Ramos, R.C.F.; Rocha, M.Â.W.; Cordeiro, M.T.; de Alencar, S.P.; Nunes, M.L. Sleep EEG patterns in infants with congenital Zika virus syndrome. *Clinical Neurophysiology* **2017**, *128*, 204-214.

9. Cauchemez, S.; Besnard, M.; Bompard, P.; Dub, T.e.; Guillemette-Artur, P.; Eyrolle-Guignot, D.; Salje, H.; Van Kerkhove, M.D.; Abadie, V.; Garel, C.*, et al.* Association between Zika virus and microcephaly in French Polynesia, 2013-15: A retrospective study. *The Lancet* **2016**, *387*, 2125-2132.

10. Cavalcanti, D.D.; Alves, L.V.; Furtado, G.J.; Santos, C.C.; Feitosa, F.G.; Ribeiro, M.C.; Menge, P.; Lira, I.M.; Alves, J.G. Echocardiographic findings in infants with presumed congenital Zika syndrome: Retrospective case series study. *Plos One* **2017**, *12*, e0175065.

11. Chibueze, E.C.; Tirado, V.; Lopes, K.d.S.; Balogun, O.O.; Takemoto, Y.; Swa, T.; Dagvadorj, A.; Nagata, C.; Morisaki, N.; Menendez, C.*, et al.* Zika virus infection in pregnancy: a systematic review of disease course and complications. *Bulll World Health Organ.* **2016**, *06*, 1-35.

12. Cragan, J.D.; Mai, C.T.; Petersen, E.E.; Liberman, R.F.; Forestieri, N.E.; Stevens, A.C.; Delaney, A.; Dawson, A.L.; Ellington, S.R.; Shapiro-mendoza, C.K.*, et al.* Baseline Prevalence of Birth Defects Associated with Congenital Zika Virus. *Morbidity and Mortality Weekly Report* **2017**, *66*, 219-220.

13. Cuevas, E.L.; Tong, V.T.; Rozo, N.; Valencia, D.; Pacheco, O.; Gilboa, S.M.; Mercado, M.; Renquist, C.M.; González, M.; Ailes, E.C.*, et al.* Preliminary Report of Microcephaly Potentially Associated with Zika Virus Infection During Pregnancy — Colombia, January–November 2016. *MMWR. Morbidity and Mortality Weekly Report* **2016**, *65*, 1409-1413.

14. Cunha, A.J.; Magalhães-Barbosa, M.C.; Lima-Setta, F.; Prata-Barbosa, A. Evolution of cases of microcephaly and neurological abnormalities suggestive of congenital infection in Brazil: 2015-2016. *Bull World Health Organ* **2016**, 1-15.

15. Cunha, A.J.L.A.d.; de Magalhães-Barbosa, M.C.; Lima-Setta, F.; Medronho, R.d.A.; Prata-Barbosa, A.; da Cunha, A.J.L.A.; de Magalhães-Barbosa, M.C.; Setta, F.L.; de Andrade Medronho, R.; Prata-Barbosa, A. Microcephaly Case Fatality Rate Associated with Zika Virus Infection in Brazil. *The Pediatric Infectious Disease Journal* **2016**, *36*, 1.

16. da Silva, A.A.M.; Ganz, J.S.S.; Sousa, P.d.S.; Doriqui, M.J.R.; Ribeiro, M.R.C.; Branco, M.d.R.d.F.C.; Queiroz, R.C.d.S.; Pacheco, M.d.J.T.; da Costa, F.R.V.; Silva, F.d.S.*, et al.* Early growth and neurologic outcomes of infants with probable congenital Zika virus syndrome. *Emerging Infectious Diseases* **2016**, *22*, 1953-1956.

17. de Araújo, T.V.B.; Rodrigues, L.C.; de Alencar Ximenes, R.A.; de Barros Miranda-Filho, D.; Montarroyos, U.R.; de Melo, A.P.L.; Valongueiro, S.; de Albuquerque, M.d.F.P.M.; Souza, W.V.; Braga, C.*, et al.* Association between Zika virus infection and microcephaly in Brazil, January to May, 2016: Preliminary report of a case-control study. In *The Lancet Infectious Diseases*, 2016; Vol. 16, pp 1356-1363.

18. de Brito, C.A.A.; de Brito, C.C.M.; Oliveira, A.C.; Rocha, M.; Atanásio, C.; Asfora, C.; Matos, J.D.; Lima, A.S.; Albuquerque, M.F.M. Zika in Pernambuco: Rewriting the first outbreak. *Revista da Sociedade Brasileira de Medicina Tropical* **2016**, *49*, 553-558.

19. De Carvalho, N.S.; De Carvalho, B.F.; Fugaça, C.A.; Dóris, B.; Biscaia, E.S. Zika virus infection during pregnancy and microcephaly occurrence: A review of literature and Brazilian data. In *Brazilian Journal of Infectious Diseases*, 2016; Vol. 20, pp 282-289.

20. de Fatima Vasco Aragao, M.; van der Linden, V.; Brainer-Lima, A.M.; Coeli, R.R.; Rocha, M.A.; Sobral da Silva, P.; Durce Costa Gomes de Carvalho, M.; van der Linden, A.; Cesario de Holanda, A.; Valenca, M.M. Clinical features and neuroimaging (CT and MRI) findings in presumed Zika virus related congenital infection and microcephaly: retrospective case series study. *Bmj* **2016**, *353*, i1901.

21. Kleber de Oliveira, W.; Cortez-Escalante, J.; De Oliveira, W.T.G.H.; do Carmo, G.M.I.; Henriques, C.M.P.; Coelho, G.E.; Araújo de França, G.V. Increase in Reported Prevalence of Microcephaly in Infants Born to Women Living in Areas with Confirmed Zika Virus Transmission During the First Trimester of Pregnancy - Brazil, 2015. *MMWR. Morbidity and mortality weekly report* **2016**, *65*, 242-247.

22. de Paula Freitas, B.; de Oliveira Dias, J.R.J.; Prazeres, J.; Sacramento, G.A.G.; Ko, A.I.; Maia, M.M.; Belfort, R.J. Ocular Findings in Infants With Microcephaly Associated With Presumed Zika Virus Congenital. *JAMA Ophthalmology* **2016**, *134*, 529-535.

23. Duijster, J.W.; Goorhuis, A.; van Genderen, P.J.J.; Visser, L.G.; Koopmans, M.P.; Reimerink, J.H.; Grobusch, M.P.; van der Eijk, A.A.; van den Kerkhof, J.H.C.T.; Reusken, C.B.*, et al.* Zika virus infection in 18 travellers returning from Surinam and the Dominican Republic, The Netherlands, November 2015-March 2016. *Infection* **2016**, *44*, 797-802.

24. Eickmann, S.H.; Carvalho, M.D.C.G.; Ramos, R.C.F.; Rocha, M.Â.W.; Linden, V.v.d.; Silva, P.F.S.d. Síndrome da infecção congênita pelo vírus Zika. *Cadernos de Saúde Pública* **2016**, *32*, 1-3.

25. Ellington, S.R.; Devine, O.; Bertolli, J.; Martinez Quiñones, A.; Shapiro-Mendoza, C.K.; Perez-Padilla, J.; Rivera-Garcia, B.; Simeone, R.M.; Jamieson, D.J.; Valencia-Prado, M.*, et al.* Estimating the Number of Pregnant Women Infected With Zika Virus and Expected Infants With Microcephaly Following the Zika Virus Outbreak in Puerto Rico, 2016. *JAMA Pediatrics* **2016**, *30341*, 1-6.

26. França, G.V.A.; Schuler-Faccini, L.; Oliveira, W.K.; Henriques, C.M.P.; Carmo, E.H.; Pedi, V.D.; Nunes, M.L.; Castro, M.C.; Serruya, S.; Silveira, M.F.*, et al.* Congenital Zika virus syndrome in Brazil: a case series of the first 1501 livebirths with complete investigation. *Lancet (London, England)* **2016**, *388*, 891-897.

27. Frieden, T.R.; Schuchat, A.; Petersen, L.R.; L, A.; SA, R.; GVA, F.; O, P.; OD, L. Zika Virus 6 Months Later. *Jama* **2016**, *374*, 1981-1987.

28. Garcia, E.; Yactayo, S.; Nishino, K.; Millot, V.; Perea, W.; Briand, S. Zika virus infection: global update on epidemiology and potentially associated clinical manifestations. In *Weekly Epidemiological Record*, 2016; Vol. 91, pp 73-81.

29. Halai, U.; Nielsen-Saines, K.; Moreira, M.; Sequeira, P.; Pereira Junior, J.; Zin, A.; Cherry, J.; Gabaglia, C.; Gaw, S.; Adachi, K.*, et al.* Maternal Zika Virus Disease Severity, Virus Load, Prior Dengue Antibodies and their Relationship to Birth Outcomes. *Clinical Infectious Diseases* **2017**.

30. Jaenisch, T.; Rosenberger, K.D.; Brito, C.; Brady, O.; Brasil, P.; Marques, E.T. Risk of microcephaly after Zika virus infection in Brazil, 2015 to 2016. *Bulletin of the World Health Organization* **2017**, *95*, 191-198.

31. Johansson, M.A.; Mier-Y-Teran-Romero, L.; Reefhuis, J.; Gilboa, S.M.; Hills, S.L. Zika and the Risk of Microcephaly. *The New England journal of medicine* **2016**, *375*, 1-4.

32. Journel, I.; Andrécy, L.L.; Metellus, D.; Pierre, J.S.; Faublas, R.M.; Juin, S.; Dismer, A.M.; Fitter, D.L.; Neptune, D.; Laraque, M.J.*, et al.* Transmission of Zika Virus — Haiti, October 12, 2015–September 10, 2016. *MMWR. Morbidity and Mortality Weekly Report* **2017**, *66*, 172-176.

33. Krauer, F.; Riesen, M.; Reveiz, L.; Oladapo, O.T.; Martínez-Vega, R.; Porgo, T.V.; Haefliger, A.; Broutet, N.J.; Low, N. Zika Virus Infection as a Cause of Congenital Brain Abnormalities and Guillain-Barré Syndrome: Systematic Review. *PLoS medicine* **2017**, *14*, e1002203.

34. Kumar, M.; Ching, L.; Astern, J.; Lim, E.; Stokes, A.J.; Melish, M.; Nerurkar, V.R. Prevalence of Antibodies to Zika Virus in Mothers from Hawaii Who Delivered Babies with and without Microcephaly between 2009-2012. *PLOS Neglected Tropical Diseases* **2016**, *10*, e0005262.

35. Ladhani, S.N.; O'Connor, C.; Kirkbride, H.; Brooks, T.; Morgan, D. Outbreak of Zika virus disease in the Americas and the association with microcephaly, congenital malformations and Guillain–Barré syndrome. *Archives of Disease in Childhood* **2016**, *0*, archdischild-2016-310590.

36. Leal, M.C.; Muniz, L.F.; Ferreira, T.S.A.; Santos, C.M.; Almeida, L.C.; Van Der Linden, V.; Ramos, R.C.F.; Rodrigues, L.C.; Neto, S.S.C. Hearing Loss in Infants with Microcephaly and Evidence of Congenital Zika Virus Infection — Brazil, November 2015–May 2016. *MMWR. Morbidity and Mortality Weekly Report* **2016**, *65*, 917-919.

37. Magalhães-Barbosa, M.C.d.; Prata-Barbosa, A.; Robaina, J.R.; Raymundo, C.E.; Lima-Setta, F.; Cunha, A.J.L.A.d. Prevalence of microcephaly in eight south-eastern and midwestern Brazilian neonatal intensive care units: 2011–2015. *Archives of Disease in Childhood* **2017**, archdischild-2016-311541.

38. Magalhães-Barbosa, M.C.d.; Prata-Barbosa, A.; Robaina, J.R.; Raymundo, C.E.; Lima-Setta, F.; Cunha, A.J.L.A.d. Trends of the microcephaly and Zika virus outbreak in Brazil, January-July 2016. *Travel Medicine and Infectious Disease* **2016**, *14*, 458-463.

39. Melo, A.S.d.O.; Aguiar, R.S.; Amorim, M.M.R.; Arruda, M.B.; Melo, F.d.O.; Ribeiro, S.T.C.; Batista, A.G.M.; Ferreira, T.; Dos Santos, M.P.; Sampaio, V.V.*, et al.* Congenital Zika Virus Infection: Beyond Neonatal Microcephaly. *JAMA neurology* **2016**, *73*, 1-10.

40. Meneses, J.d.A.; Ishigami, A.C.; de Mello, L.M.; Albuquerque, L.L.d.; Brito, C.A.A.d.; Tenório Cordeiro, M.; Pena, L.J. Lessons Learned at the Epicenter of Brazil's Congenital Zika Epidemic: Evidence from 87 Confirmed Cases. *Clinical Infectious Diseases* **2017**.

41. Millichap, J.G. Zika Virus Infection and Microcephaly. *Pediatric Neurology Briefs* **2016**, *30*, 8.

42. Miranda, H.A.d.; Costa, M.C.; Frazão, M.A.M.; Simão, N.; Franchischini, S.; Moshfeghi, D.M. Expanded Spectrum of Congenital Ocular Findings in Microcephaly with Presumed Zika Infection. *Ophthalmology* **2016**, *123*, 1788-1794.

43. Moore, C.A.; Staples, J.E.; Dobyns, W.B.; Pessoa, A.; Ventura, C.V.; Fonseca, E.B.d.; Ribeiro, E.M.; Ventura, L.O.; Neto, N.N.; Arena, J.F.*, et al.* Characterizing the Pattern of Anomalies in Congenital Zika Syndrome for Pediatric Clinicians. *JAMA Pediatrics* **2016**, *171*, 1-8.

44. Morris, J.K.; Rankin, J.; Garne, E.; Loane, M.; Greenlees, R.; Addor, M.-C.; Arriola, L.; Barisic, I.; Bergman, J.E.H.; Csaky-Szunyogh, M.*, et al.* Prevalence of microcephaly in Europe: population based study. *BMJ (Clinical research ed.)* **2016**, *354*, i4721.

45. Nishiura, H.; Mizumoto, K.; Rock, K.S.; Yasuda, Y.; Kinoshita, R.; Miyamatsu, Y. A theoretical estimate of the risk of microcephaly during pregnancy with Zika virus infection. *Epidemics* **2016**, *15*, 66-70.

46. Nunes, M.L.; Carlini, C.R.; Marinowic, D.; Neto, F.K.; Fiori, H.H.; Scotta, M.C.; Zanella, P.L.v.; Soder, R.B.; Da Costa, J.C. Microcephaly and Zika virus: A clinical and epidemiological analysis of the current outbreak in Brazil. In *Jornal de Pediatria*, 2016; Vol. 92, pp 230-240.

47. de Oliveira, W.K.; Carmo, E.H.; Henriques, C.M.; Coelho, G.; Vazquez, E.; Cortez-Escalante, J.; Molina, J.; Aldighieri, S.; Espinal, M.A.; Dye, C. Zika Virus Infection and Associated Neurologic Disorders in Brazil. *New England Journal of Medicine* **2017**, *376*, NEJMc1608612.

48. Paixão, E.S.; Barreto, F.; Da Glória Teixeira, M.; Da Conceição N Costa, M.; Rodrigues, L.C. History, epidemiology, and clinical manifestations of Zika: A systematic review. *American Journal of Public Health* **2016**, *106*, 606-612.

49. Paploski, I.A.D.; Prates, A.P.P.B.; Cardoso, C.W.; Kikuti, M.; Silva, M.M.O.; Waller, L.A.; Reis, M.G.; Kitron, U.; Ribeiro, G.S. Time lags between exanthematous illness attributed to Zika virus, Guillain-Barré Syndrome, and Microcephaly, Salvador, Brazil. *Emerging Infectious Diseases* **2016**, *22*, 1438-1444.

50. Rasmussen, S.A.; Jamieson, D.J.; Honein, M.A.; Petersen, L.R. Zika Virus and Birth Defects — Reviewing the Evidence for Causality. *New England Journal of Medicine* **2016**, *374*, 1-7.

51. Reefhuis, J.; Gilboa, S.M.; Johansson, M.A.; Valencia, D.; Simeone, R.M.; Hills, S.L.; Polen, K.; Jamieson, D.J.; Petersen, L.R.; Honein, M.A. Projecting month of birth for At-Risk infants after zika virus disease outbreaks. *Emerging Infectious Diseases* **2016**, *22*, 828-832.

52. Saad-Roy, C.M.; van den Driessche, P.; Ma, J. Estimation of Zika virus prevalence by appearance of microcephaly. *BMC Infectious Diseases* **2016**, *16*, 1-6.

53. Schuler-Faccini, L.; Ribeiro, E.M.; Feitosa, I.M.L.; Horovitz, D.D.G.; Cavalcanti, D.P.; Pessoa, A.; Doriqui, M.J.R.; Neri, J.I.; Neto, J.M.d.P.; Wanderley, H.Y.C.*, et al.* Possible Association Between Zika Virus Infection and Microcephaly - Brazil, 2015. *MMWR. Morbidity and mortality weekly report* **2016**, *65*, 59-62.

54. Simeone, R.M.; Shapiro-Mendoza, C.K.; Meaney-Delman, D.; Petersen, E.E.; Galang, R.R.; Oduyebo, T.; Rivera-Garcia, B.; Valencia-Prado, M.; Newsome, K.B.; Pérez-Padilla, J.*, et al.* Possible Zika Virus Infection Among Pregnant Women — United States and Territories, May 2016. *MMWR. Morbidity and Mortality Weekly Report* **2016**, *65*, 514-519.

55. Simões, R.; Buzzini, R.; Bernardo, W.; Cardoso, F.; Salomão, A.; Cerri, G. Zika virus infection and pregnancy. *Revista da Associacao Medica Brasileira (1992)* **2016**, *62*, 108-115.

56. Souza, W.V.d.; Araújo, T.V.B.d.; Albuquerque, M.d.F.P.M.; Braga, M.C.; Ximenes, R.A.d.A.; Miranda-Filho, D.d.B.; Bezerra, L.C.A.; Dimech, G.S.; Carvalho, P.I.d.; Assunção, R.S.d.*, et al.* Microcephaly in Pernambuco State, Brazil: epidemiological characteristics and evaluation of the diagnostic accuracy of cutoff points for reporting suspected cases. *Cadernos de saude publica* **2016**, *32*, e00017216.

57. Teixeira, M.G.; Da Conceição N Costa, M.; De Oliveira, W.K.; Nunes, M.L.; Rodrigues, L.C. The epidemic of Zika virus-related microcephaly in Brazil: Detection, control, etiology, and future scenarios. *American Journal of Public Health* **2016**, *106*, 601-605.

58. Thomas, D.L.; Sharp, T.M.; Torres, J.; Armstrong, P.A.; Munoz-Jordan, J.; Ryff, K.R.; Martinez-Quiñones, A.; Arias-Berríos, J.; Mayshack, M.; Garayalde, G.J.*, et al.* Local Transmission of Zika Virus — Puerto Rico, November 23, 2015–January 28, 2016. *MMWR. Morbidity and Mortality Weekly Report* **2016**, *65*, 154-158.

59. van der Linden, V.; Pessoa, A.; Dobyns, W.; Barkovich, A.J.; Júnior, H.v.d.L.; Filho, E.L.R.; Ribeiro, E.M.; Leal, M.d.C.; Coimbra, P.P.d.A.; Aragão, M.d.F.V.V.*, et al.* Description of 13 Infants Born During October 2015–January 2016 With Congenital Zika Virus Infection Without Microcephaly at Birth — Brazil. *MMWR. Morbidity and Mortality Weekly Report* **2016**, *65*, 1343-1348.

60. Vargas, A.; Saad, E.; Dimech, G.S.; Santos, R.H.; Sivini, M.A.V.C.; Albuquerque, L.C.; Lima, P.M.S.; Barreto, I.d.C.; Andrade, M.E.d.; Estima, N.M.*, et al.* Características dos primeiros casos de microcefalia possivelmente relacionados ao vírus Zika notificados na Região Metropolitana de Recife, Pernambuco. *Epidemiologia e Serviços de Saúde* **2016**, *25*, 691-700.

61. Ventura, C.V.; Maia, M.; Travassos, S.B.; Martins, T.T.; Patriota, F.; Nunes, M.E.; Agra, C.; Torres, V.L.; van der Linden, V.; Ramos, R.C.*, et al.* Risk Factors Associated With the Ophthalmoscopic Findings Identified in Infants With Presumed Zika Virus Congenital Infection. *JAMA Ophthalmology* **2016**, *134*, 912-918.

62. Ventura, C.V.; Fernandez, M.P.; Gonzalez, I.A.; Rivera-Hernandez, D.M.; Lopez-Alberola, R.; Peinado, M.; Floren, A.A.; Rodriguez, P.A.; Williams, B.K.; de la Vega Muns, G.*, et al.* First Travel-Associated Congenital Zika Syndrome in the US: Ocular and Neurological Findings in the Absence of Microcephaly. *Ophthalmic surgery, lasers & imaging retina* **2016**, *47*, 952-955.

63. Ventura, C.V.; Ventura, L.O.; Bravo-Filho, V.; Martins, T.T.; Berrocal, A.M.; Gois, A.L.; de Oliveira Dias, J.R.; Araújo, L.; Escarião, P.; van der Linden, V.*, et al.* Optical Coherence Tomography of Retinal Lesions in Infants With Congenital Zika Syndrome. *JAMA Ophthalmology* **2016**, *062*, 1-8.

64. Ventura, C.V.; Maia, M.; Ventura, B.V.; Van Der Linden, V.; Araújo, E.B.; Ramos, R.C.; Rocha, M.A.W.; Carvalho, M.D.C.G.; Belfort, R.; Ventura, L.O. Ophthalmological findings in infants with microcephaly and presumable intra-uterus Zika virus infection. *Arquivos Brasileiros de Oftalmologia* **2016**, *79*, 1-3.

65. Ventura, L.O.; Ventura, C.V.; Lawrence, L.; van der Linden, V.; van der Linden, A.; Gois, A.L.; Cavalcanti, M.M.; Barros, E.A.; Dias, N.C.; Berrocal, A.M.*, et al.* Visual impairment in children with congenital Zika syndrome. *Journal of American Association for Pediatric Ophthalmology and Strabismus* **2017**, *0*.

66. Villamil-Gómez, W.E.; Mendoza-Guete, A.; Villalobos, E.; González-Arismendy, E.; Uribe-García, A.M.; Castellanos, J.E.; Rodríguez-Morales, A.J. Diagnosis, management and follow-up of pregnant women with Zika virus infection: A preliminary report of the ZIKERNCOL cohort study on Sincelejo, Colombia. *Travel Medicine and Infectious Disease* **2016**, *14*, 155-158.

67. Walker, W.L.; Lindsey, N.P.; Lehman, J.A.; Krow-Lucal, E.R.; Rabe, I.B.; Hills, S.L.; Martin, S.W.; Fischer, M.; Staples, J.E. Zika Virus Disease Cases - 50 States and the District of Columbia, January 1-July 31, 2016. *MMWR. Morbidity and mortality weekly report* **2016**, *65*, 983-986.
