# Supplementary material for: Microcephaly Prevalence in Infants Born to Zika Virus-Infected Women: A Systematic Review and Meta-Analysis
Source: Int J Mol Sci. 2017 Aug 5;18(8):1714. doi: 10.3390/ijms18081714 (PMC5578104; doi:10.3390/ijms18081714)
Supplement: Supplementary file 1 [file ijms-18-01714-s001.zip › Supplementary Table 1.docx]

*Supplementary Table 1 legend.*

| **Question** | **Question statement** |
| --- | --- |
| 1 | Did the study address a clearly focused issue? |
| 2 | Was the cohort recruited in an acceptable way? |
| 3 | Was the exposure accurately measured to minimise bias? |
| 4 | Was the outcome accurately measured to minimise bias? |
| 5(a) | Have the authors identified all important confounding factors? |
| 5(b) | Have they taken account of the confounding factors in the design and/or analysis? |
| 6(a) | Was the follow up of subjects complete enough? |
| 6(b) | Was the follow up of subjects long enough? |
| 7 | What are the results of this study? |
| 8 | How precise are the results? |
| 9 | Do you believe the results? |
| 10 | Can the results be applied to the local population? |
| 11 | Do the results of this study fit with other available evidence? |
| 12 | What are the implications of this study for practice? |

Supplementary Table 1. Responses to the Critical Appraisal Skills Programme (CASP) cohort study checklist [1]

| **Reference** | **Question** | **Response** |
| --- | --- | --- |
| [2] | 1 | Yes |
|  | 2 | Yes |
|  | 3 | Yes |
|  | 4 | Yes |
|  | 5(a) | Yes |
|  | 5(b) | Yes |
|  | 6(a) | Yes |
|  | 6(b) | No |
|  | 7 | Nine women presented Zika virus infection, resulting in two miscarriages and one case of severe microcephaly |
|  | 8 | No statistics analyses are provided (descriptive study) |
|  | 9 | Yes |
|  | 10 | Yes |
|  | 11 | Yes |
|  | 12 | Zika virus infection during pregnancy was associated with a range of outcomes and prompted the creation of a CDC registry of Zika virus infection among pregnant women |
| [3] | 1 | Yes |
|  | 2 | Yes |
|  | 3 | No |
|  | 4 | Yes |
|  | 5(a) | Yes |
|  | 5(b) | Yes |
|  | 6(a) | No |
|  | 6(b) | No |
|  | 7 | The autors found four cases of microcephaly among 1484 pregnancies from women with confirmed Zika virus infection |
|  | 8 | The authors estimated Zika virus incidence and female-to-male cases ratio, and the results seem fair and precise |
|  | 9 | Yes |
|  | 10 | Yes |
|  | 11 | Yes |
|  | 12 | The study adds evidence that Zika virus infection during the third trimester is less associated with negative pregnancy outcomes |

| **Reference** | **Question** | **Response** |
| --- | --- | --- |
| [4] | 1 | Yes |
|  | 2 | Yes |
|  | 3 | Yes |
|  | 4 | Yes |
|  | 5(a) | Yes |
|  | 5(b) | Yes |
|  | 6(a) | Yes |
|  | 6(b) | Yes |
|  | 7 | The authors reported four microcephaly cases, and observed a spectrum of central nervous system abnormalities |
|  | 8 | The authors conducted comparative analyses of Zika-virus positive pregnant women with Zika virus-negative pregnant women, and the data seem fair, but the nature of analyses did not elicited confidence interval calculation |
|  | 9 | Yes |
|  | 10 | Yes |
|  | 11 | Yes |
|  | 12 | Zika-virus exposed children should be reassessed periodically for neurodevelopmental effects, since negative outcomes following infection was substantial |
| [5] | 1 | Yes |
|  | 2 | Yes |
|  | 3 | Yes |
|  | 4 | Can’t tell |
|  | 5(a) | Yes |
|  | 5(b) | Can’t tell |
|  | 6(a) | Yes |
|  | 6(b) | Can’t tell |
|  | 7 | The authors conducted immunosurveilance of Zika virus infection and found no cases of microcephaly among 65 pregnancies resulting from a 32% prevalence of Zika virus infection (16522 individuals enrolled in total) |
|  | 8 | The authors conducted observational, descriptive research, therefore there were no comparative statistical analysis, but the results seem fair |
|  | 9 | Yes |
|  | 10 | Yes |
|  | 11 | Yes |
|  | 12 | Mosquito control and prevention are important, and individuals with recent travel to Zika-affected areas should consider testing after their return |

| **Reference** | **Question** | **Response** |
| --- | --- | --- |
| [6] | 1 | Yes |
|  | 2 | Yes |
|  | 3 | Yes |
|  | 4 | Yes |
|  | 5(a) | Yes |
|  | 5(b) | Yes |
|  | 6(a) | Yes |
|  | 6(b) | Yes |
|  | 7 | The authors found a prevalence of microcephaly around 6% |
|  | 8 | The results seem fair and precise, since the reported 95% CI values for the prevalence of microcephaly are close to other estimates in the literature |
|  | 9 | Yes |
|  | 10 | Yes |
|  | 11 | Yes |
|  | 12 | The results support the importance of Zika virus infection screening during pregnancy |
| [7] | 1 | Yes |
|  | 2 | Yes |
|  | 3 | Yes |
|  | 4 | Yes |
|  | 5(a) | Can’t tell |
|  | 5(b) | Can’t tell |
|  | 6(a) | Yes |
|  | 6(b) | Yes |
|  | 7 | The authors found a prevalence of microcephaly around 2% |
|  | 8 | The authors conducted comparative analyses of Zika-virus positive pregnant women with Zika virus-negative pregnant women, and the data seem fair |
|  | 9 | Yes |
|  | 10 | Yes |
|  | 11 | Yes |
|  | 12 | The authors state that it is paramount to evaluate suspected cases by detailed neurosonographic examination on a monthly basis, paying particular attention to the corpus callosum and the presence of hyperechogenic foci |

| **Reference** | **Question** | **Response** |
| --- | --- | --- |
| [8] | 1 | Yes |
|  | 2 | Yes |
|  | 3 | Yes |
|  | 4 | Yes |
|  | 5(a) | Yes |
|  | 5(b) | Yes |
|  | 6(a) | Yes |
|  | 6(b) | Yes |
|  | 7 | The authors found a prevalence of microcephaly around 7% |
|  | 8 | The results seem fair and precise, since the reported 95% CI values for the prevalence of microcephaly are close to other estimates in the literature |
|  | 9 | Yes |
|  | 10 | Yes |
|  | 11 | Yes |
|  | 12 | The proportion of fetuses and infants with Zika virus– associated birth defects was highest among those with first trimester Zika virus infections, and therefore women who might get pregnant should avoid mosquito exposure |
| [9] | 1 | Yes |
|  | 2 | Yes |
|  | 3 | Yes |
|  | 4 | Yes |
|  | 5(a) | Can’t tell |
|  | 5(b) | Yes |
|  | 6(a) | Yes |
|  | 6(b) | Yes |
|  | 7 | The authors observed no infants with microcephaly |
|  | 8 | The results seem fair and precise, but 95% CI were not calculated due to the study being descriptive |
|  | 9 | Yes |
|  | 10 | Yes |
|  | 11 | Yes |
|  | 12 | The authors state that knowledge of the baseline prevalence of symptomatic and asymptomatic Zika virus infection in pregnant travelers and the travel characteristics of at-risk women will be important as the global epidemic worsens; they identified that 4% were at risk from reported travel with only 2/1000 infected. |

**References**

1. Critical Appraisal Skills Programme. CASP Cohort Study Checklist. Available online: <http://www.casp-uk.net/checklists> (acessed on 1 June 2017).

2. Meaney-Delman, D.; Hills, S.L.; Williams, C.; Galang, R.R.; Iyengar, P.; Hennenfent, A.K.; Rabe, I.B.; Panella, A.; Oduyebo, T.; Honein, M.A.*, et al.* Zika Virus Infection Among U.S. Pregnant Travelers - August 2015-February 2016. *MMWR. Morbidity and mortality weekly report* **2016**, *65*, 211-214.

3. Pacheco, O.; Beltrán, M.; Nelson, C.a.; Valencia, D.; Tolosa, N.; Farr, S.L.; Padilla, A.V.; Tong, V.T.; Cuevas, E.L.; Espinosa-Bode, A.*, et al.* Zika Virus Disease in Colombia - Preliminary Report. *The New England journal of medicine* **2016**, 1-10.

4. Brasil, P.; Pereira, J., Jose P.; Raja Gabaglia, C.; Damasceno, L.; Wakimoto, M.; Ribeiro Nogueira, R.M.; Carvalho de Sequeira, P.; Machado Siqueira, A.; Abreu de Carvalho, L.M.; Cotrim da Cunha, D.*, et al.* Zika Virus Infection in Pregnant Women in Rio de Janeiro — Preliminary Report. *New England Journal of Medicine* **2016**, *375*, NEJMoa1602412.

5. Adams, L.; Bello-Pagan, M.; Lozier, M.; Ryff, K.R.; Espinet, C.; Torres, J.; Perez-Padilla, J.; Febo, M.F.; Dirlikov, E.; Martinez, A.*, et al.* Update: Ongoing Zika Virus Transmission - Puerto Rico, November 1, 2015-July 7, 2016. *MMWR. Morbidity and mortality weekly report* **2016**, *65*, 774-779.

6. Honein, M.A.; Dawson, A.L.; Petersen, E.E.; Jones, A.M.; Lee, E.H.; Yazdy, M.M.; Ahmad, N.; Macdonald, J.; Evert, N.; Bingham, A.*, et al.* Birth Defects Among Fetuses and Infants of US Women With Evidence of Possible Zika Virus Infection During Pregnancy. *JAMA* **2016**, *317*, 59.

7. Pomar, L.; Malinger, G.; Benoist, G.; Carles, G. Association between Zika virus and foetopathy: a prospective cohort study in French Guiana. Preliminary report. *Ultrasound Obstet Gynecol.* **2017**, 1-22.

8. Reynolds, M.R.; Jones, A.M.; Petersen, E.E.; Lee, E.H.; Rice, M.E.; Bingham, A.; Ellington, S.R.; Evert, N.; Reagan-Steiner, S.; Oduyebo, T.*, et al.* Vital Signs: Update on Zika Virus–Associated Birth Defects and Evaluation of All U.S. Infants with Congenital Zika Virus Exposure — U.S. Zika Pregnancy Registry, 2016. *MMWR. Morbidity and Mortality Weekly Report* **2017**, *66*, 366-373.

9. Adhikari, E.H.; Nelson, D.B.; Johnson, K.A.; Jacobs, S.; Rogers, V.L.; Roberts, S.; Sexton, T.; McIntire, D.D.; Casey, B. Infant outcomes among women with Zika virus infection during pregnancy: Results of a large prenatal Zika screening program. *American Journal of Obstetrics and Gynecology* **2017**, *216*, 292.e291-292.e298.
